# Associations Between Bed Bugs and Mental Illness Among Emergency Department Patients

**DOI:** 10.7759/cureus.15024

**Published:** 2021-05-14

**Authors:** Johnathan M Sheele

**Affiliations:** 1 Emergency Medicine, Mayo Clinic, Jacksonville, USA

**Keywords:** anxiety, bed bug, cimex lectularius, depression, insomnia, alcohol, psychosis, suicidality, bedbug, abuse

## Abstract

Background

Bed bugs are common urban pests associated with stress, anxiety, depression, and some reports of suicidality. The emergency department (ED) treats patients with both acute mental illness and bed bug infestations. There have been few studies examining associations between bed bug infestations and mental illness.

Methods

A case-control study involving 332 adult ED patients infested with bed bugs and 4,952 uninfested control patients matched on sex, age (±1 year at the time of the ED visit), and the specific ED was completed. All clinic encounters occurred in northeast Ohio between February 1, 2011, and February 1, 2017, from a single health system. Univariable and multivariable regression analysis looked for associations between bed bug infestation and different psychiatric diagnoses and medications.

Results

Bed bug infested patients were more likely than uninfested patients to screen positive for an unsafe home and needing an abuse consult at ED triage (*P*≤.03 for both). ED psychiatric evaluations were not significantly more common among those with (2.4%) and without (1.3%) bed bugs (*P*=.14). Bed bug infested patients were significantly more likely to have an ED or inpatient diagnosis of alcohol abuse and psychosis (*P*≤.03 for both), but not for depression or suicidality. On univariable analysis, among ED patients not admitted to the hospital, bed bug infested patients were more likely to be diagnosed with psychosis or schizophrenia/schizoaffective disease (P≤.02) than uninfested patients. Among ED patients that received an ED or inpatient psychiatric evaluation and were admitted to the hospital, bed bug infested patients were significantly less likely to be diagnosed with depression and suicidality (*P*≤.03 for both). However, they were not significantly more likely to have insomnia or anxiety.

Discussion

Among ED patients, bed bug infestations were not associated with an ED or inpatient diagnosis of depression or suicidality. On univariable analysis, some mental health diagnoses such as anxiety and insomnia were more common among ED patients with bed bugs, but these associations were no longer significant on multivariable analysis. These associations likely reflect the complex relationships between socioeconomic factors, health disparities, mental illness, and having a bed bug infestation.

## Introduction

Bed bugs are hematophagous ectoparasitic pests that can be especially problematic in urban environments. *Cimex lectularius* L, known as the common bed bug, and *Cimex hemipterus*, known as the tropical bed bug, are common bed bug species that feed on humans. Bed bugs affect all socioeconomic backgrounds, and infestations have not always correlated with property values [1]. However, it is generally believed that the most vulnerable members of society - those living in urban environments, persons who are internally displaced, persons with mental illness, and persons with limited income - are at the highest risk for a bed bug infestation and are the least capable of adapting to and eliminating the infestation [2-7]. A low income, less education, and a higher unemployment rate have higher real and perceived concerns for getting bed bugs [7-9]. Obtaining used clothing and furniture, living in group homes and apartment complexes, and staying at homeless shelters may be additional risk factors coming into contact with bed bugs [7,9-11].

Persons who live in poverty and in urban areas can have higher rates of preexisting mental health conditions and distress from increased anxiety and stress [12]. Bed bugs can exacerbate a person’s stress and adversely affect the quality of life, and they may cause and exacerbate an existing mental health disease [13]. Few research or epidemiologic investigations have evaluated the psychological effects of bed bugs, and most of what has been published are anecdotal [5,14]. However, some published reports suggest that bed bug infestations are associated with insomnia and sleep disturbances, anxiety, mood changes, panic, agitation, phobias, delusions, social withdrawal, suicide, depression, and post-traumatic stress disorder-like symptoms [5,14-17]. In contrast, other reports show no association between insomnia and depression related to bed bug infestations [7,9,18]. Although little is known about the relationship between bed bug infestation and substance abuse, bed bugs have not been shown to be associated with alcohol consumption [19]. However, the stress of an infestation could unmask or exacerbate a preexisting substance abuse disorder [16,17].

Poor and mentally ill persons may have a harder time eliminating an infestation because they may lack financial resources and social support [5,17]. Additionally, the fear of bed bug reinfestation may result in maladaptive behaviors, such as hypervigilance, insomnia, unreasonable precautions to prevent reinfection, and exacerbation of social isolation [17]. The objectives of the present study were to determine whether bed bug infested patients evaluated in the emergency department (ED) were more likely to have a diagnosis of or be treated for a psychiatric disorder.

## Materials and methods

Institutional review board approval was obtained from University Hospitals (UH). UH information technology personnel obtained data from the UH electronic health records of ED patients treated at one of nine UH hospitals or free-standing EDs in northeast Ohio. All patients were adults (age ≥ 18 years) and were treated between February 1, 2011, and February 1, 2017. Searches of the electronic health record using the keywords “bedbug,” “bed bug,” “Cimex,” or “lectularius” were performed as there is no International Classification of Diseases code specific to bed bugs. The principal investigator then reviewed clinical encounters and identified 332 patients in which a bed bug infestation was reported by the patient or suspected by clinical staff, or a bed bug was found on the patient during the clinical encounter. Bed bug infested patients were matched 1:15 to control patients with no documented history of bed bugs. Control patients were matched based on sex, age (±1 year at the time of the ED visit), and the specific ED. Only the first ED visit where bed bugs were identified was included in the dataset. The first recorded triage and laboratory testing set were used in the present analysis. All current ED diagnoses, inpatient diagnoses, and past diagnoses were provided in text format, which was then searched using keyword searches in Excel (Microsoft Corp). Keyword searches were used to define suicidality, any psychiatric condition, any sleep-related condition, anxiety, psychosis, alcohol misuse, and illicit drug use (Table 1). The analysis did not include 28 patients who died in the ED. Manuscripts have been previously published from the dataset [19,20].

**Table 1:** Text Search Terms Used for Diagnoses

Diagnosis	Text search terms
Bipolar:	bipolar
Insomnia:	insomnia
Suicidality:	suicide attempt or suicidal
Any psychiatric condition:	suicide, suicidal, bipolar, psychosis, schizophrenia, depression, depressive disorder, anxiety, hallucinations, schizoaffective, post-traumatic stress disorder, PTSD, abuse, panic attack, eating disorder, personality disorder, psychotic, delusion, mania, major depressive disorder, premenstrual dysphoric disorder, hoarder, hoarding, obsessive-compulsive disorder, trichotillomania, acute stress disorder, factitious disorder, anorexia nervosa, bulimia nervosa, binge eating disorder, kleptomania, addiction, borderline personality, antisocial personality, agoraphobia, panic attack, panic disorder, separation anxiety, adjustment disorder, dissociative amnesia, dissociative identity disorder, multiple personality disorder, depersonalization disorder, derealization disorder, conversion disorder, Factitious disorder, Munchausen syndrome, delusional parasitosis, Morgellons, rumination disorder, pica, binge-eating disorder, pyromania, Intermittent explosive disorder, oppositional defiant disorder, disruptive mood dysregulation disorder, disorganized speech, paranoia, paranoid, bizarre behavior, body-dysmorphic disorder, skin picking, excoriation disorder, avoidant personality disorder, histrionic personality disorder, schizoid, or schizotypal
Any sleep-related condition:	narcolepsy, hypersomnolence, parasomnias, insomnia, restless leg syndrome, periodic limb movement disorder, REM sleep behavior disorder, parasomnias, sleep-related movement disorders, obstructive sleep apnea, or upper airway resistance syndrome
Anxiety:	anxiety, panic attack, or panic disorder
Depression:	depression or depressive disorder
Schizophrenia or schizoaffective:	schizophrenia or schizoaffective
Psychosis:	psychosis, psychotic, hallucinations, delusional, or delusion
Alcohol abuse:	alcohol use or abuse, alcoholism, or alcohol-related disorder
Illicit drug abuse:	polysubstance, substance abuse, alcohol, alcoholism, intoxication, intoxicated, tobacco, cigarette, cocaine, heroin, marijuana, methamphetamine, PCP, narcotic abuse, opioid abuse, phencyclidine, alcohol-related disorder, cannabis-related disorder, inhalant-use disorder, or nicotine

Statistical analysis

Categorical variables were summarized with frequency (percentage) and were compared using the Fisher exact test for 2×2 analysis or the Chi-squared test. Continuous variables were summarized with a mean (standard deviation [SD]) and were compared with the use of the t-test. A forward stepwise regression approach was used to identify significant variables (P<.05) for multivariable regression analysis. Unless otherwise stated, all regression analyses were performed accounting for age, race (Black/African American vs [versus] other), history of tobacco use identified at ED triage, or as a past or current medical diagnosis (yes vs no/unknown), mechanism of ED arrival, and the patient’s location before ED arrival (home; nursing or rehabilitation facility; physician office, clinic, surgery center, or inpatient elsewhere), marital status, any current or past diagnosis of alcohol misuse, ED triage abuse screen for an unsafe home, and admission to the hospital from the ED. All tests were two sided, and P-values ≤.05 were considered statistically significant. The statistical analysis was performed with JMP Pro 14 software (SAS Institute Inc., Cary, NC, USA).

## Results

The analysis evaluated 332 patients with bed bugs and 4,952 patients without bed bugs. Baseline patient characteristics are summarized in Table 2.

**Table 2:** Characteristics of Emergency Department Patients %, percentage; ED, emergency department; EMS, emergency medical service; N, number; y, year

	Patients		
Characteristic	Bed bugs, N (%) (N=332)	No bed bugs, N (%) (N=4,952)	P-value
Age, y			≥ .99
≤40	47 (14.2)	703 (14.2)	
41-68	171 (51.5)	2,548 (51.5)	
≥69	114 (34.3)	1,701 (34.3)	
Race			< .001
Other than Black/African American	57 (17.2)	2,233 (45.1)	
Black/African American	274 (82.5)	2,684 (54.2)	
Marital status			< .001
Married/life partner	47 (14.2)	1,659 (33.5)	
Single	190 (57.2)	1,990 (40.2)	
Divorced/separated/widowed	92 (27.7)	1,262 (25.5)	
Health insurance			.02
Medicare	98 (29.5)	1,322 (26.7)	
Medicaid	26 (7.8)	229 (4.6)	
Unknown/no insurance	31 (9.3)	452 (9.1)	
Private	177 (53.3 )	2,949 (59.6)	
Location before ED visit			< .001
Home	323 (97.3)	4,442 (89.7)	
Rehabilitation or nursing facility	5 (1.5)	140 (2.8)	
Physician office/surgery center/clinic/inpatient at another acute care facility	2 (0.6)	244 (4.9)	
Discharged from ED	68 (20.5)	3,117 (62.9)	< .001
Mechanism of ED arrival			< .001
Private vehicle	98 (29.5)	3,036 (61.3)	
EMS, helicopter, or police transport	166 (50.0)	1,655 (33.4)	
Public transportation, walked, or other	68 (20.5)	261 (5.3)	

The majority of patients with bed bugs were Black or African American race (56.4%), single (57.2%), and female (57.1%). Patients with bed bugs were less likely to be discharged home from the ED and less likely to arrive at the ED by private vehicle. No significant difference was observed in mean (SD) blood alcohol levels between those with bed bugs (236.8 [SD, 111.0] g/dL) (n=10) and those without bed bugs (228.7 [SD, 159.4] g/dL) (n=97) (P=.78).

Triage

On univariable analysis, the bed bug infested patients had higher heart rates (89.7 vs 84.4 beats/minute [min]), had higher respiratory rates (18.8 vs 18.1 breaths/min), reported more pain (4.7 vs 3.8 [scale, 0-10]), and were less likely to have a primary care physician (28.9% vs 39.6%) (P≤.02 for all) (Table 3). ED triage screening questions identified that bed bug infested patients were more likely to have homicidal ideation, screen positive for an unsafe home, and need an abuse consult than those without bed bugs (P≤.006 for all). On multivariable regression analysis, bed bug infested patients had higher heart rates, higher triage pain scores, and higher emergency severity index scores; they screened positive for an unsafe home and needed an abuse consult (P≤.03 for all).

**Table 3:** Emergency Department Triage and Medications Given to Patients ^a^Values are presented as the number (%) of patients unless specified otherwise. %, percentage; CI, confidence interval; ED, emergency department; ESI, emergency severity index; F, Fahrenheit; min, minute; mmHg, millimeters per mercury; n, number; NA, not applicable; OR, odds ratio; SD, standard deviation; SpO_2_, peripheral capillary oxygen saturation

	Patients					
Characteristics	Bed bugs^a ^(n=332)	No bed bugs^a ^(n=4,952)	Unadjusted OR (95% CI)	P-value	Adjusted OR (95% CI)	Adjusted P-value
Triage, mean (SD)						
Temperature, °F	97.7 (1.31)	97.8 (1.15) (n=4,890)	NA	.31	0.97 (0.86-1.11)	.69
Heart rate, beats/min	89.7 (16.41) (n=331)	84.4 (17.63) (n=4,900)	NA	< .001	1.01 (1.00-1.02)	.03
Respiratory rate, breaths/min	18.8 (4.88) (n=331)	18.1 (2.74) (n=4,888)	NA	.02	1.00 (0.96-1.05)	.88
Mean arterial pressure, mmHg	101.8 (22.01) (n=330)	102.4 (17.60) (n=4,891)	NA	.60	0.99 (0.99-1.00)	.17
Peripheral capillary oxygen saturation (SpO_2_), %	97.6 (2.51) (n=331)	97.6 (2.63) (n=4,884)	NA	> .99	0.99 (0.10-3.92)	.63
Triage pain scale (score, 0-10)	4.7 (3.67) (n=176)	3.8 (3.59) (n=1,430)	NA	.003	1.08 (1.02-1.14)	.01
ESI (scale, 1-5)	2.9 (0.81) (n=323)	2.9 (0.74) (n=4,751)	NA	.34	1.81 (1.39-2.36)	< .001
Has a primary care physician	96 (28.9) (n=332)	1,961 (39.6) (n=4,952)	0.62 (0.49-0.79)	< .001	0.83 (0.60-1.16)	.28
Triage screen positive for suicidal ideation (vs no/unknown)	5 (1.7) (n=294)	41 (0.9) (n=4,569)	1.91 (0.75-4.87)	.20	0.32 (0.07-1.49)	.15
Triage screen positive for homicidal ideation (vs no/unknown)	6 (2.0) (n=297)	22 (0.5) (n=4,623)	4.31 (1.73-10.72)	.006	1.05 (0.27-4.03)	.94
Triage screen positive for depression (vs no/unknown)	16 (6.0) (n=268)	119 (4.0) (n=2,949)	1.51 (0.88-2.58)	.15	0.63 (0.32-1.26)	.19
Triage screen positive for depression, suicidal, or homicidal ideation (vs no/unknown to all)	22 (6.6) (n=332)	147 (3.0) (n=4,952)	2.32 (1.46-3.68)	< .001	0.67 (0.36-1.26)	.22
Triage screen positive for suicidal or homicidal ideation (vs no/unknown to all)	9 (3.1) (n=294)	55 (1.2) (n=4,565)	2.59 (1.27-5.29)	.01	0.68 (0.24-1.94)	.47
Received psychiatric evaluation in ED or as inpatient	41 (12.4) (n=332)	130 (2.6) (n=4,952)	5.23 (3.61-7.57)	< .001	1.35 (0.77-2.39)	.29
Triage chronic pain screen	53 (23.1) (n=229)	273 (18.4) (n=1,488)	1.34 (0.96-1.87)	.09	1.28 (0.86-1.91)	.22
Triage abuse screen unsafe at home	14 (5.3) (n=296)	34 (1.2) (n=2,935)	4.74 (2.51-8.95)	< .001	3.71 (1.74-7.90)	< .001
Triage abuse consult needed	29 (11.6) (n=250)	47 (2.9) (n=1,638)	4.44 (2.74-7.21)	< .001	2.44 (1.22-4.91)	.01
Drug treatment administered in ED						
Antipsychotic (quetiapine, haloperidol, ziprasidone, aripiprazole, olanzapine, risperidone, lurasidone, or clozapine) administered in ED	6 (1.8) (n=332)	37 (0.8) (n=4,948)	2.44 (1.02-5.83)	.05	0.74 (0.15-3.73)	.71
Benzodiazepine (lorazepam, diazepam, clonazepam, or alprazolam) administered in ED	19 (5.7) (n=332)	222 (4.5) (n=4,948)	1.29 (0.80-2.09)	.28	0.89 (0.46-1.71)	.72

Medications

On univariable analysis, antipsychotic medications were more frequently administered to bed bug infested patients in the ED (1.8% vs .8%) (P=.05), but this association was no longer significant on regression analysis (Table 3). Benzodiazepine use was not more frequent in bed bug infested patients.

ED and inpatient diagnosis

On univariable analysis, bed bug infested patients were significantly more likely than patients without bed bugs to have a current ED or inpatient diagnosis of bipolar disorder, insomnia, any psychiatric condition, any sleep-related condition, anxiety, schizophrenia or schizoaffective disorder, psychosis, alcohol misuse, and illicit drug use (P≤.02 for all) (Table 4). Patients with bed bugs were not more likely to have a current ED or inpatient diagnosis of depression or have suicidality than those without bugs. On multivariable regression analysis, those with bed bugs were significantly more likely to have a current ED or inpatient diagnosis of psychosis and alcohol misuse (P≤.03 for both).

**Table 4:** Patients With an Emergency Department or Inpatient Psychiatric Diagnosis ^a^Regression analysis did not include a current or past diagnosis of a psychiatric illness. ^b^Regression analysis did not include the past or current alcohol abuse variable. %, percentage; CI, confidence interval; ED, emergency department; N, number; OR, odds ratio

	Patients					
Diagnosis	Bed bugs, N (%) (N=332)	No bed bugs, N (%) (N=4,952)	Unadjusted OR (95% CI)	P-value	Adjusted OR ^a^(95% CI)	Adjusted P-value^a^
Bipolar disorder (n=81)	12 (3.6)	69 (1.4)	2.65 (1.42-4.95)	.004	1.95 (0.70-5.45)	.20
Insomnia (n=117)	14 (4.2)	103 (2.1)	2.07 (1.17-3.66)	.02	1.60 (0.77-3.33)	.21
Suicidality (n=55)	5 (1.5)	50 (1.0)	1.50 (0.59-3.78)	.39	0.21 (0.04-1.02)	.05
Any psychiatric condition (n=986)	124 (37.3)	862 (17.4)	2.83 (2.24-3.58)	< .001	1.26 (0.90-1.76)	.18
Any sleep-related condition (n=322)	33 (9.9)	289 (5.8)	1.78 (1.22-2.60)	.004	0.96 (0.59-1.55)	.87
Anxiety (n=299)	29 (8.7)	270 (5.5)	1.66 (1.11-2.48)	.02	1.39 (0.83-2.33)	.21
Depression (n=318)	28 (8.4)	290 (5.9)	1.48 (0.99-2.22)	.07	0.98 (0.58-1.65)	.93
Schizophrenia or schizoaffective disorder (n=102)	25 (7.5)	77 (1.6)	5.16 (3.23-8.21)	< .001	1.83 (0.89-3.75)	.10
Psychosis (n=83)	19 (5.7)	64 (1.3)	4.64 (2.74-7.83)	< .001	2.78 (1.32-5.87)	.007
Alcohol misuse^b^ (n=217)	43 (13.0)	174 (3.5)	4.09 (2.87-5.82)	< .001	1.76 (1.07-2.89)	.03
Illicit drug use (n=136)	33 (9.9)	103 (2.1)	5.20 (3.45-7.82)	< .001	1.15 (0.62-2.12)	.66

When only the patients admitted from the ED were examined (Table 5), multiple diagnoses were observed on univariable analysis that were associated significantly with bed bug infestation, including bipolar disorder, any psychiatric condition, schizophrenia or schizoaffective disorder, psychosis, alcohol misuse, and illicit drug use (P≤.003 for all). However, on regression analysis, the only significant association was that bed bug infested patients were less likely to have suicidality (P=.04).

**Table 5:** Admitted Emergency Department Patients Regression analysis does not include ED disposition %, percentage; CI, confidence interval; ED, emergency department; N, number; OR, odds ratio

	Bed bugs % (N=265)	No bed bugs % (N=1856)	OR (95% CI)	p-value	Adjusted OR (95% CI)	Adjusted P-value
Bipolar	4.2% (11)	1.4% (25)	3.17 (1.54-6.52)	.003	1.25 (.42-3.76)	.69
Insomnia	5.3% (14)	3.2% (59)	1.70 (.93-3.09)	.10	1.74 (.82-3.69)	.15
Suicidality	1.5% (4)	1.7% (31)	.90 (.32-2.58)	1.00	.20 (.04-.92)	.04
Any psychiatric condition	41.9% (111)	27.3% (506)	1.92 (1.48-2.51)	< .001	.78 (.21-2.86)	.71
Any sleep-related disorder	12.5% (33)	11.2% (207)	1.13 (.77-1.68)	.53	.99 (.61-1.61)	.97
Anxiety	9.4% (25)	9.3% (172)	1.02 (.66-1.58)	.91	1.17 (.64-2.14)	.62
Depression	8.7% (23)	9.3% (173)	.92 (.59-1.46)	.82	.80 (.44-1.46)	.47
Schizophrenia or schizoaffective	7.9% (21)	2.4% (45)	3.46 (2.03-5.91)	< .001	1.26 (.58-2.74)	.57
Psychosis	6.0% (16)	2.4% (44)	2.64 (1.47-4.76)	.002	1.91 (.85-4.29)	.12
Alcohol abuse	15.1% (40)	5.3% (99)	3.16 (2.13-4.67)	< .001	1.59 (.17-14.81)	.68
Illicit drug use	11.7% (31)	3.1% (58)	4.11 (2.60-6.49)	< .001	1.14 (.59-2.17)	.70

Among only the patients who were not admitted from the ED, patients with bed bugs were significantly more likely to have a diagnosis of schizophrenia or schizoaffective disorder, any psychiatric disorder, and psychosis (P≤.04 for all) (Table 6). No significant differences were detected on multivariable analysis.

**Table 6:** Non-admitted Emergency Department Patients Regression analysis does not include emergency department disposition %, percentage; CI, confidence interval; ED, emergency department; N, number; NA, not applicable; OR, odds ratio

	Bed bugs % (N=68)	No bed bugs % (N=3117)	OR (95% CI)	P-value	Adjusted OR (95% CI)	Adjusted P-value
Bipolar	1.5% (1)	1.6% (49)	.93 (.13-6.87)	1.00	.49 (.05-5.04)	.55
Insomnia	0% (0)	1.4% (45)	NA	1.00	NA	NA
Suicidality	1.5% (1)	.9% (28)	1.65 (.22-12.28)	.47	NA	NA
Any psychiatric condition	20.6% (14)	12.0% (373)	1.91 (1.05-3.47)	.04	NA	NA
Any sleep-related disorder	0% (0)	2.7% (84)	NA	.26	NA	NA
Anxiety	5.9% (4)	3.3% (103)	1.83 (.65-5.12)	.29	1.52 (.30-7.71)	.61
Depression	7.4% (5)	4.1% (128)	1.85 (.73-4.69)	.21	.54 (.09-3.21)	.50
Schizophrenia or schizoaffective	5.9% (4)	1.2% (38)	5.06 (1.76-14.61)	.01	2.30 (.36-14.67)	.38
Psychosis	4.4% (3)	.8% (25)	5.71 (1.68-19.38)	.02	3.09 (.45-21.20)	.25
Alcohol abuse	4.4% (3)	2.4% (75)	1.87 (.57-6.09)	.23	NA	NA
Illicit drug use	2.9% (2)	1.5% (48)	1.94 (.46-8.14)	.29	NA	NA

New ED and inpatient diagnoses

Bed bug infestation was associated with a new ED or inpatient diagnosis (i.e., the patient had no documented past history of the diagnosis) of bipolar disorder, insomnia, suicidality, any psychiatric condition, anxiety, depression, schizophrenia or schizoaffective disorder, alcohol misuse, and illicit drug use (P≤.04 for all) (Table 7). The infestation also was associated with a new diagnosis of psychosis, psychotic disorder, hallucinations, delusional disorder, or delusion (P≤.04). On multivariable regression analysis, patients with bed bugs had bipolar disorder, any psychiatric condition, anxiety, schizophrenia or schizoaffective disorder, and psychosis (P≤.02 for all).

**Table 7:** New Emergency Department or Inpatient Diagnosis (No Documented Past History of the Diagnosis) %, percentage; CI, confidence interval; ED, emergency department; N, number; OR, odds ratio

	Patients					
Psychiatric diagnosis	Bed bugs, N (%) (N=332)	No bed bugs, N (%) (N=4,952)	OR (95% CI)	P-value	Adjusted OR (95% CI)	Adjusted P-value
Bipolar	6 (1.8)	10 (0.2)	9.10 (3.29-25.18)	< .001	30.18 (5.39-168.89)	< . 001
Insomnia	4 (1.2)	17 (0.3)	3.54 (1.18-10.58)	.04	2.14 (0.42-11.00)	.36
Suicide attempt or suicidal ideation	5 (1.5)	20 (0.4)	3.77 (1.41-10.11)	.02	0.28 (0.05-1.41)	.12
Any psychiatric condition	44 (13.3)	163 (3.3)	4.49 (3.15-6.39)	< .001	2.15 (1.34-3.46)	.002
Any sleep-related disorder	5 (1.5)	46 (0.9)	1.63 (0.64-4.13)	.25	0.96 (0.28-3.31)	.94
Anxiety, panic attack, or panic disorder	9 (2.7)	47 (0.9)	2.91 (1.41-5.99)	.008	2.98 (1.18-7.51)	.02
Depression or depressive disorder	11 (3.3)	52 (1.1)	3.23 (1.67-6.25)	.002	2.08 (0.87-4.96)	.10
Schizophrenia or schizoaffective disorder	9 (2.7)	12 (0.2)	11.47 (4.80-27.42)	< .001	4.82 (1.37-16.94)	.01
Psychosis, psychotic disorder, hallucinations, delusional disorder, or delusion	17 (5.1)	25 (0.5)	10.64 (5.68-19.90)	< .001	2.15 (1.34-3.46)	.002
Current or past diagnosis of alcohol use or misuse, alcoholism, or alcohol-related disorder	9 (2.7)	29 (0.6)	4.73 (2.22-10.08)	< .001	1.70 (0.49-5.89)	.40
Any illicit drug use	8 (2.4)	17 (0.3)	7.17 (3.07-16.73)	< .001	1.08 (0.36-3.25)	.89

Among patients who received an ED or inpatient psychiatric evaluation, bed bug infested patients were significantly more likely to receive a new diagnosis of bipolar disorder, any psychiatric condition, and psychosis (P≤.04 for all) (Table 8).

**Table 8:** New Emergency Department (ED) or Inpatient Diagnosis (No Documented Past History of the Diagnosis) and Received an ED or Inpatient Psychiatric Evaluation %, percentage; CI, confidence interval; ED, emergency department; N, number; OR, odds ratio

	Patients			
Diagnosis	Bed bugs, N (%) (N=40)	No bed bugs, N (%) (N=124)	OR (95% CI)	P-value
Bipolar	4 (10.0)	0 (0.0)		.003
Insomnia	1 (2.5)	1 (0.8)	3.15 (0.19-51.61)	.43
Suicide attempt or suicidal ideation	4 (10.0)	15 (12.1)	0.81 (0.25-2.59)	> .99
Any psychiatric condition	10 (25.0)	13 (10.5)	2.85 (1.14-7.13)	.03
Any sleep-related disorder	1 (2.5)	1 (0.8)	3.15 (0.19-51.61)	.43
Anxiety, panic attack, or panic disorder	3 (7.5)	5 (4.0)	1.93 (0.44-8.46)	.40
Depression or depressive disorder	4 (10.0)	8 (6.5)	1.61 (0.46-5.66)	.49
Schizophrenia or schizoaffective disorder	4 (10.0)	4 (3.2)	3.33 (0.79-14.0)	.10
Psychosis, psychotic disorder, hallucinations, delusional disorder, or delusion	8 (20.0)	10 (8.1)	2.85 (1.04-7.82)	.04
Current or past diagnosis of alcohol use or abuse, alcoholism, or alcohol-related disorder	1 (2.5)	2 (1.6)	1.56 (0.14-17.72)	.57
Any illicit drug use	1 (2.5)	4 (3.2)	0.77 (0.08-7.09)	> .99

Psychiatric evaluation

Bed bug infested patients were not more likely to receive an ED psychiatric evaluation than matched uninfested ED patients (2.4% vs 1.3%; P=.14). Bed bug infested patients who received an ED or inpatient psychiatric evaluation were significantly more likely to be admitted to the hospital, use illicit drugs, and have schizophrenia or schizoaffective disorder than those without bed bugs (P≤.05 for all) (Table 9). They also were less likely to screen positive for depression in ED triage and to have suicidal ideation (P≤.02). On multivariable regression analysis, the only significant variable between patients with and those without bed bugs was that infested patients were less likely to screen positive for depression in ED triage (P=.02).

**Table 9:** Data for ED or Inpatient Psychiatric Evaluation ^a^History of any psychiatric disease not included in regression analysis %, percentage; CI, confidence interval; ED, emergency department; N, number; NA, not applicable; OR, odds ratio; vs, versus

	Patients					
Evaluation	Bed bugs, N (%) (N=40)	No bed bugs, N (%) (N=124)	OR (95% CI)	P-value	Adjusted OR (95% CI)	Adjusted P-value
Admitted	38 (95.0)	76 (61.3)	12.00 (2.77-52.04)	< .001	NA	NA
Drug treatment administered in the ED						
Antipsychotic medication (quetiapine, haloperidol, ziprasidone, aripiprazole, olanzapine, risperidone, lurasidone, or clozapine) administered in ED	5 (12.5)	15 (12.1)	1.04 (0.35-3.06)	> .99	2.18 (0.15-32.78)	.57
Benzodiazepine (lorazepam, diazepam, clonazepam, or alprazolam) administered in ED	7 (17.5)	42 (33.9)	0.41 (0.17-1.01)	.07	0.51 (0.10-2.61)	.42
Triage screen						
Positive for suicidal ideation (vs no/unknown)	4 (12.5) (n=32)	31 (29.0) (n=107)	0.35 (0.11-1.08)	.07	0.21 (0.03-1.66)	.14
Positive for homicidal ideation (vs no/unknown)	4 (12.5) (n=32)	15 (14.2) (n=106)	0.87 (0.27-2.82)	> .99	0.33 (0.04-2.74)	.30
Positive for depression (vs no/unknown)	4 (15.4) (n=26)	43 (44.8) (n=96)	0.22 (0.07-0.70)	.006	0.13 (0.02-0.70)	.02
ED/inpatient diagnosis						
Bipolar disorder	7 (17.5)	14 (11.3)	1.67 (0.62-4.47)	.41	1.21 (0.23-6.42)	.83
Anxiety, panic attack, panic disorder	8 (20.0)	34 (27.4)	0.66 (0.28-1.58)	.41	1.19 (0.28-5.11)	.82
Depression, depressive disorder	9 (22.5)	50 (40.3)	0.43 (0.19-0.98)	.06	0.31 (0.06-1.57)	.16
Insomnia	3 (7.5)	6 (4.8)	1.59 (0.38-6.69)	.69	1.76 (0.20-15.20)	.61
Any sleep-related disorder	6 (15.0)	13 (10.5)	1.51 (0.53-4.27)	.41	0.89 (0.17-4.59)	.89
Suicide attempt or suicidal ideation	4 (10.0)	35 (28.2)	0.28 (0.09-0.85)	.02	NA	NA
Alcohol use or misuse, alcoholism, or alcohol-related disorder	9 (22.5)	26 (21.0)	1.09 (0.46-2.58)	.83	NA	NA
Illicit drug use	13 (32.5)	21 (16.9)	2.36 (1.04-5.31)	.04	1.72 (0.41-7.14)	.45
Any psychiatric condition	36 (90.0)	108 (87.1)	1.33 (0.42-4.25)	.78	NA	NA
Schizophrenia or schizoaffective disorder	14 (35.0)	23 (18.6)	2.36 (1.07-5.22)	.049	NA	NA
Psychosis	10 (25.0)	29 (23.4)	1.09 (0.48-2.50)	.83	0.91 (0.22-3.82)	.90

Hospital admission

Among those patients admitted to the hospital and who received an ED or inpatient psychiatric evaluation, the only significant difference between those with and without bed bugs was less likely for those with bed bugs to have suicidality and depression (P≤.03 for both) (Table 10).

**Table 10:** Emergency Department or Inpatient Psychiatric Evaluation and Admitted to the Hospital %, percentage; CI, confidence interval; ED, emergency department; N, number; OR, odds ratio

	Bed bugs % (N=39)	No bed bugs % (N=98)	OR (95% CI)	P-value
Bipolar	18.0% (7)	13.3% (13)	1.43 (.52-3.91)	.59
Insomnia	7.7% (3)	5.1% (5)	1.55 (.35-6.82)	.69
Suicidality	7.7% (3)	30.6% (30)	.19 (.05-.66)	.004
Any psychiatric condition	89.7% (35)	87.7% (86)	1.22 (.37-4.05)	1.00
Any sleep-related disorder	15.4% (6)	12.2% (12)	1.30 (.45-3.76)	0.59
Anxiety	18.0% (7)	29.6% (29)	.52 (.21-1.31)	.20
Depression	18.0% (7)	37.8% (37)	.36 (.14-.90)	.03
Schizophrenia or schizoaffective	33.3% (13)	17.4% (17)	2.38 (1.02-5.55)	.07
Psychosis	23.1% (9)	23.5% (23)	.98 (.41-2.36)	1.00
Alcohol abuse	23.1% (9)	20.4% (20)	1.17 (.48-2.86)	.82
Illicit drug use	33.3% (13)	19.4% (19)	2.08 (.90-4.78)	.12

## Discussion

Bed bug infested patients were not more likely to receive an ED psychiatric evaluation than those without bed bugs. However, bed bug infested patients were significantly more likely to be admitted to the hospital and receive an ED or inpatient psychiatric evaluation. Among admitted patients receiving an ED or inpatient psychiatric evaluation, new psychiatric diagnoses were not more common than those without bed bugs. While patients with bed bugs may be depressed about the infestation or the inability to eliminate it, they were not more likely to have an ED or inpatient diagnosis of depression or suicidality. These findings contrast with previous case reports and other investigations; however, they did not focus on ED patients [16,17].

Fleas and head lice cause disturbed sleep, but reports are conflicting about whether bed bugs are associated with insomnia [5,7,9,14,21,22]. In the current study, on univariable analysis but not on multivariable analysis, patients with bed bugs were more likely to have an ED and inpatient diagnosis of a sleep-related condition and insomnia. Bed bugs may cause insomnia and disturbed sleep in some infested persons, but this analysis only involved ED patients. ED and inpatient clinicians likely did not explore a sleep-related condition unless it was pertinent to the reason for the clinical visit.

Patients may feel anxiety about having bed bugs, an effect that has been shown for other ectoparasites [5,23-25]. Bed bug infested patients were significantly more likely to have an ED or inpatient diagnosis of anxiety univariable but not multivariable analysis. This finding could be a reflection of the specific ED patient population that was studied.

Bed bugs are not definitively known to be associated with substance use [16,18]. Bed bug infested patients were more likely to receive a diagnosis in the ED or the inpatient setting of an alcohol-related disorder or illicit drug use. Still, these associations were not significant on multivariable regression analysis. Additionally, no significant differences were observed in mean blood alcohol levels between those with and without bed bugs.

Limitations

There are no formal screening or triage processes at UH to evaluate patients with bed bugs, and not all ED patients were likely screened for having bed bug infestations. It may not be safe to assess critically ill, altered, demented, psychotic, and violent patients for bed bugs; however, when a bed bug is found on a patient it is documented by health care providers. The two-step method (i.e., UH IT data extraction and then manual chart review) to identify bed bug cases was intended to minimize miscategorizing patients without bed bugs as having bed bugs. The burden of confirmed bed bug infestations (e.g., the insect was found on the patient) among patients at the UH tertiary care ED has been well documented and occurs about ~5 days [7,9,26-28]. Additionally, patients could have been misclassified as having bed bugs due to acquiring the insects in an ambulance or while waiting in the ED, but these occurrences are believed to be unlikely based on previous research at UH [7,9,26-28].

All patients in our study presented to a regular ED for evaluation, there were no separate free-standing psychiatric-only EDs. Emergency medicine clinicians likely did not specifically evaluate the patients' mental health unless it was pertinent to their reason for coming to the ED. Therefore it cannot be determined if bed bugs might be related to milder adverse mental health effects or have psychological effects unrelated to presenting to the ED. No international classification of disease (e.g., International Classification of Disease) has a code specific for bed bug infestation, and all diagnoses in the present dataset were provided in text format that required keyword searches. The keyword searches could have miscategorized patients as having or not having a condition including bed bugs. The provided diagnoses do not include all psychiatric diseases or conditions, especially when a patient presented to the ED with a nonpsychiatric condition. In addition, the dataset did not include results of specific psychiatric assessments, physical examination, or urine drug screens. Patients who transferred to non-UH facilities for a formal psychiatric evaluation or hospital admission would not have those diagnoses available for this analysis.

## Conclusions

On univariable analysis, bed bug infested patients in the ED were significantly more likely to receive ED or inpatient diagnoses of bipolar disorder, insomnia, anxiety, schizophrenia or schizoaffective disorder, psychosis, alcohol misuse, and illicit drug use, but only alcohol misuse and psychosis remained significant on multivariable analysis. Previous reports have identified depression and suicidality among some patients with bed bug infestations, but this study did not find that depression and suicidality were not more common among bed bug infested ED patients than matched uninfested ED patients.
